# Study protocol of the SACURA trial: a randomized phase III trial of efficacy and safety of UFT as adjuvant chemotherapy for stage II colon cancer

**DOI:** 10.1186/1471-2407-12-281

**Published:** 2012-07-07

**Authors:** Megumi Ishiguro, Hidetaka Mochizuki, Naohiro Tomita, Yasuhiro Shimada, Keiichi Takahashi, Kenjiro Kotake, Masahiko Watanabe, Yukihide Kanemitsu, Hideki Ueno, Toshiaki Ishikawa, Hiroyuki Uetake, Shigeyuki Matsui, Satoshi Teramukai, Kenichi Sugihara

**Affiliations:** 1 Department of Surgical Oncology, Tokyo Medical and Dental University, Graduate School, 1-5-45 Yushima, Bunkyo-ku, Tokyo, 113-8519, Japan; 2 Department of Surgery, National Defense Medical College, 3-2 Namiki, Tokorozawa, Saitama, 359-8513, Japan; 3 Department of Surgery, Hyogo College of Medicine, 1-1 Mukogawa-cho, Nishinomiya, Hyogo, 663-8501, Japan; 4 Division of Gastrointestinal Medical Oncology, National Cancer Center Hospital, 5-1-1 Tsukiji, Chuo-ku, Tokyo, 104-0045, Japan; 5 Department of Surgery, Cancer and Infectious Diseases Center Komagome Hospital, 18-22, Honkomagome 3-chome, Bunkyo-ku, Tokyo, 113-8677, Japan; 6 Department of Surgery, Tochigi Cancer Center, 4-9-13 Yonan, Utsunomiya, Tochigi, 320-0834, Japan; 7 Department of Surgery, Kitasato University School of Medicine, 1-15-1 Kitasato, Minami-ku, Sagamihara, Kanagawa, 252-0375, Japan; 8 Department of Gastroenterological Surgery, Aichi Cancer Center Hospital, 1-1 Kanokoden, Chikusa-ku, Nagoya, Aichi, 464-8681, Japan; 9 Department of Translational Oncology, Tokyo Medical and Dental University, Graduate School, 1-5-45 Yushima, Bunkyo-ku, Tokyo, 113-8519, Japan; 10 Department of Data Science, the Institute of Statistical Mathematics, 10-3 Midori-cho, Tachikawa, Tokyo, 190-8562, Japan; 11 Department of Clinical Trial Design and Management, Translational Research Center, Kyoto University Hospital, 54 Shogoin-kawaharacho, Sakyo-ku, Kyoto, 606-8507, Japan

**Keywords:** Colon cancer, Stage II, Adjuvant chemotherapy, UFT, Risk factor, Predictive factor, Prognostic factor, Surgery-alone, Randomized controlled trial, Japan

## Abstract

**Background:**

Adjuvant chemotherapy for stage III colon cancer is internationally accepted as standard treatment with established efficacy, but the usefulness of adjuvant chemotherapy for stage II colon cancer remains controversial. The major Western guidelines recommend adjuvant chemotherapy for “high-risk stage II” cancer, but this is not clearly defined and the efficacy has not been confirmed.

**Methods/design:**

SACURA trial is a multicenter randomized phase III study which aims to evaluate the superiority of 1-year adjuvant treatment with UFT to observation without any adjuvant treatment after surgery for stage II colon cancer in a large population, and to identify “high-risk factors of recurrence/death” in stage II colon cancer and predictors of efficacy and adverse events of the chemotherapy. Patients aged between 20 and 80 years with curatively resected stage II colon cancer are randomly assigned to a observation group or UFT adjuvant therapy group (UFT at 500–600 mg/day as tegafur in 2 divided doses after meals for 5 days, followed by 2-day rest. This 1-week treatment cycle is repeated for 1 year). The patients are followed up for 5 years until recurrence or death. Treatment delivery and adverse events are entered into a web-based case report form system every 3 months. The target sample size is 2,000 patients. The primary endpoint is disease-free survival, and the secondary endpoints are overall survival, recurrence-free survival, and incidence and severity of adverse events. In an additional translational study, the mRNA expression of 5-FU-related enzymes, microsatellite instability and chromosomal instability, and histopathological factors including tumor budding are assessed to evaluate correlation with recurrences, survivals and adverse events.

**Discussion:**

A total of 2,024 patients were enrolled from October 2006 to July 2010. The results of this study will provide important information that help to improve the therapeutic strategy for stage II colon cancer.

**Trial registration:**

ClinicalTrials.gov NCT00392899.

## Background

In Japan, colorectal cancer is the second most common cancer following stomach cancer, and the third most fatal cancer following lung cancer and stomach cancer [1]. Postoperative adjuvant chemotherapy has been demonstrated to improve the outcome in stage III colon cancer and is internationally accepted as standard treatment. On the other hand, no consensus has been reached on the usefulness of adjuvant chemotherapy for stage II colon cancer.

A meta-analysis using the studies C-01 to C-04 of the National Surgical Adjuvant Breast & Bowel Project (NSABP) [2] showed that adjuvant chemotherapy significantly decreased the risk of recurrence/death in both Dukes’ B and C. However, other pooled analysis or large population database review revealed no statistically significant additive survival benefit of adjuvant therapy including 5-FU + leucovorin exclusively in stage II colon cancer [3,4].

In Japan, Sakamoto et al. [5] reported the results of the meta-analysis that adjuvant therapy with oral 5-FU drugs (without concomitant use of leucovorin) contributed to significant improvement in recurrence-free survival (RFS) and overall survival (OS) in stage II colon cancer. UFT (Taiho Pharmaceutical Co., Ltd., Tokyo, Japan) is one of the most widely used oral 5-FU agent as adjuvant chemotherapy for colorectal cancer in Japan. UFT is a combination drug of tegafur and uracil at a molar ratio of 1:4 and is characterized by long maintenance of a high 5-FU concentration level converted from tegafur in blood/tumors due to inhibition of degeneration of 5-FU by uracil. In the randomized controlled trial (RCT) comparing 2-year adjuvant therapy using UFT (400 mg/body) with observation without adjuvant therapy in 289 patients after surgery for stage II/III colon and rectal cancer [6], the 5-year RFS was significantly better in the UFT group. However, the analysis exclusively for colon cancer (160 patients) revealed no significant difference (77.4% in the UFT group, 74.0% in the observation group, p = 0.71). In the RCT comparing 1-year adjuvant therapy using UFT (400 mg/m^2^/day) with observation without adjuvant therapy in 610 patients after surgery for stage III colon and rectal cancer [7], 1-year treatment with UFT was well tolerated and significantly improved the RFS and OS in rectal cancer, while the analysis for 332 patients with colon cancer showed no significant difference in both the 5-year RFS (71.3% in the UFT group, 69.6% in the observation group, p = 0.56) and OS.

Although both of the abovementioned two RCTs [6,7] failed to demonstrate an additive effect, 1- or 2-year postoperative adjuvant therapy with UFT alone has often been used for stage II colon cancer in clinical practice in Japan, because of its good feasibility [8] and low-cost. The Japanese Study Group for Postoperative Follow-up of Colorectal Cancer reported that the 5-year survival rate of 1,262 patients with stage II colon cancer who underwent surgery between 1977 and 2000 was 82.1% [9]. Given such a good outcome, it is necessary to clarify in a larger population whether postoperative adjuvant treatment with UFT alone has an additive effect on stage II colon cancer compared with observation only.

On the other hand, the reports using a large-scale database disclosed that stage II colon cancer included subpopulations with different prognosis [9,10]. The major Western guidelines recommended to select the “high-risk group of recurrence” in stage II colon cancer and to give postoperative adjuvant chemotherapy. The NCCN guidelines of 2012 [11] lists T4 lesions, number of lymph-nodes examined <12, perforation, lymphovascular involvement, poorly differentiated histopathology, and perineural invasion as high-risk factors, while the ASCO guidelines of 2004 [12] lists inadequately sampled nodes, T4 lesions, perforation, and poorly differentiated histology as factors for considering for adjuvant chemotherapy in stage II colon cancer. In addition to these, high CEA is listed as high-risk factor in the ESMO guidelines [13]. Recently, the biomarker studies have proposed new risk factors for recurrence/prognosis.

It seems appropriate to use adjuvant chemotherapy for a subgroup with poor prognosis in stage II colon cancer. However, the definition of “high-risk stage II” is not clear yet, and the efficacy of adjuvant chemotherapy for those patients has not been demonstrated. We therefore conducted the SACURA trial (Surgical Adjuvant Chemotherapy with UFT for Curatively Resected Stage II Colon Cancer), a multicenter phase III RCT to verify the efficacy of adjuvant chemotherapy for curatively resected stage II colon cancer in a large population through evaluating the superiority of 1-year adjuvant treatment with UFT to observation without any adjuvant treatment, and to identify “high-risk factors of recurrence” in stage II colon cancer and predictors of efficacy and adverse events (AEs) of the chemotherapy.

## Methods/design

### The design of study

**Figure 1:**
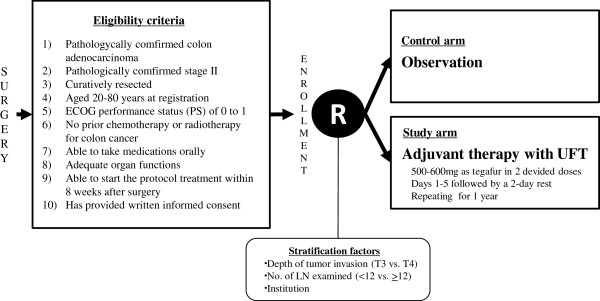
Study schema.

This study is a multicenter randomized phase III trial, in which patients with curatively resected stage II colon cancer are randomly assigned to either the observation group or UFT adjuvant therapy group (Figure 1). The primary endpoint is disease-free survival (DFS), and the secondary endpoints are OS, RFS, and incidence and severity of AEs. Superiority of adjuvant therapy with UFT compared to observation without any adjuvant therapy is evaluated. As an additional translational study, the surgical specimens are collected for histopathological and biomolecular assessments.

### Enrollment and allocation

Eligible patients are enrolled at the Translational Research Informatics Center using a web-based system. Patients are randomly assigned, in a 1:1 ratio, to either an observation group or UFT adjuvant therapy group, using minimization by introducing a random element with a 0.8 assignment probability [14], balanced on the following stratification factors: depth of tumor invasion (T3 vs. T4), number of lymph-nodes examined (<12 vs. ≥12) and institution (Figure 1). Treatment assignment is not masked from the investigators and patients.

The main eligibility criteria are as follows:

### *Inclusion criteria*

1) Histologically confirmed stage II colon cancer

2) Histologically confirmed adenocarcinoma

3) Has undergone curative surgery

4) Age: 20–80 years

5) ECOG performance status: 0–1

6) No prior chemotherapy or radiotherapy for colon cancer

7) Able to take medications orally

8) Adequate organ functions as listed below (at ≤14 days prior to enrollment)

i) Leukocytes: 3,500/mm^3^ to 12,000/mm^3^

ii) Neutrophil: ≥ 1,500/mm^3^

iii) Hemoglobin ≥ 9.0 g/dL

iv) Platelet count ≥ 100,000/mm³

v) Total bilirubin ≤ 2.0 mg/dL

vi) Aspartate aminotransferase (AST), alanine aminotransferase (ALT): ≤ 100 IU/L

vii) Creatinine: ≤1.5 mg/dL

9) Able to start the protocol treatment within 8 weeks after surgery

10) Has provided written informed consent

### *Exclusion criteria*

1) Other active malignancies (i.e. diagnosed within 5 years) (Tis colorectal cancers are allowed to enroll)

2) Hereditary colorectal cancer

3) Severe comorbidities:

i) Severe postoperative complication

ii) Uncontrollable diabetes mellitus

iii) Uncontrollable hypertension

iv) Myocardial infarction within 6 months

v) Unstable angina pectoris

vi) Cirrhosis or liver failure

vii) Interstitial pneumonia, pulmonary fibrosis, or severe emphysema

viii) Psychiatric disorder

4) Concern about pregnancy

5) The investigator considers the patient not suitable for the study

### Protocol treatment

Assigned treatment is started within 8 weeks after surgery.

#### Observation group

**Figure 2:**
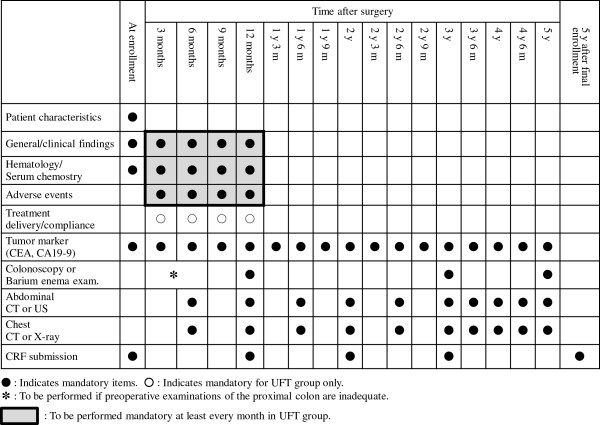
Observation, examination, and report schedule.

Patients are followed-up without adjuvant treatment, according to the schedule defined in the study protocol for 5 years until recurrence, other malignancy or death is confirmed (Figure 2).

#### UFT adjuvant therapy group

UFT is given at a dose of 500–600 mg/day as tegafur in 2 divided doses after meals for 5 days, followed by a 2-day rest [8]. This one-week cycle is repeated for one year. During protocol treatment, clinical findings and laboratory values are evaluated every month.

Protocol treatment is started and continued when the patients fulfill the following criteria: leukocytes ≥3,000/mm^3^, platelets ≥100,000/mm^3^, AST and ALT ≤100 IU/L, total bilirubin ≤2.0 mg/dL, no greater than grade 2 anorexia, nausea, vomiting, or diarrhea. If the criteria for starting/continuing treatment are not met, treatment is postponed or temporarily suspended until AEs improve to meet the criteria. And then, treatment is resumed at one dose level lower (−200 mg). The dose can be reduced if the physician judges that dose reduction is necessary. Once the dose has been reduced, it is not to be subsequently re-increased.

Protocol treatment is discontinued in the cases as follows: treatment fails to be resumed within 29 days after being postponed or temporarily suspended (the planned drug rest is not included), the physician judges that the protocol treatment is difficult to continue due to AEs, recurrence or other malignancies develop, the patient requests discontinuation of protocol treatment, and the patients withdraw informed consent.

After the completion of protocol treatment, patients are followed-up following the same schedule as for the observation group (Figure 2) until recurrence, other malignancy or death is confirmed.

### Evaluation of treatment delivery and adverse events

#### Treatment delivery (UFT adjuvant therapy group only)

Physicians report the treatment delivery via a web-based case report system, including the followings: daily dose, drug compliance*, temporary suspension (+/-), number of days of suspension, reason for suspension, dose reduction (+/-), etc.

* The drug compliance for each 3 months period is defined as the ratio of the dose actually taken to the prescribed dose, and is classified to the following 4 categories: 1) ≥90% taken, 2) ≥75% to <90% taken, 3) ≥50% to <75% taken, and 4) <50% taken.

#### Safety profile (both groups)

The types and severities of AEs from the start of protocol treatment to 30 days after the last administration are evaluated according to the National Cancer Institute Common Terminology Criteria for Adverse Events version 3.0. The most severe grade of each AE is reported every 3 months. The following AEs are required to be reported as “priority survey items”: leukocytes, hemoglobin, platelets, total bilirubin, AST, ALT, stomatitis, anorexia, nausea, vomiting, diarrhea, rash/desquamation, hyperpigmentation, and fatigue.

### Statistical background

#### Definition of endpoint

The primary endpoint of this study is DFS, and the secondary endpoints are OS, RFS, and incidence and severity of AEs. DFS is defined as the time to recurrence, other malignancies or death, whichever comes first. Patients alive and free of recurrence or other malignancies are censored at time of last follow-up. RFS is defined as the time to recurrence or death. Patients alive and free of recurrence are censored at time of last follow-up. The intervals are calculated from the date of enrollment.

#### Definition of target sample size

In two clinical studies conducted in Japanese patients with colon cancer in the 1990’s, the 5-year DFS rate in patients without adjuvant chemotherapy was 74.3% (Dukes’ B) [15] and 74.0% (Dukes’ B and C) [6]. Given a recent improved surgical outcome, it was assumed that the 5-year DFS rate would be 80% in the control group (observation group). With an expected 5-year DFS rate of 85% (hazard ratio: 0.729) in the study treatment group (UFT adjuvant therapy group), a two-sided significance level of 5%, and a power of 90%, the necessary sample size was calculated to be 970 patients per group according to the method described by Shoenfeld et al. [16]. A target sample size of 1,000 patients per group (a total of 2,000 patients in two groups) was determined in consideration of a 3% excluded rate.

#### Analysis plan

The primary analyses are done on an intent-to-treat basis. The survival curves (DFS, OS, and RFS) are estimated by the Kaplan-Meier method, and the stratified log-rank test, stratified by the depth of tumor invasion and the number of lymph-nodes examined, are used to test the null hypothesis that the respective curves are equal between the two groups. The hazard ratio is estimated using a stratified proportional hazard model. A two-sided significance level of 5% is used. Subgroup analyses are performed according to sex, age, depth of tumor invasion, and number of lymph-nodes examined for comparison between the two groups.

The treatment delivery in the UFT adjuvant therapy group is summarized. The incidence of AEs between two groups is compared with the Fisher’s exact test.

An interim analysis of the efficacy is planned at 3 years after enrollment of the last patient. For the primary endpoint (DFS), the significant levels in interim and final analyses are determined according to α spending function (the O’Brien-Fleming type) to keep the overall type I error at 5%.

### Additional translational study

**Figure 3:**
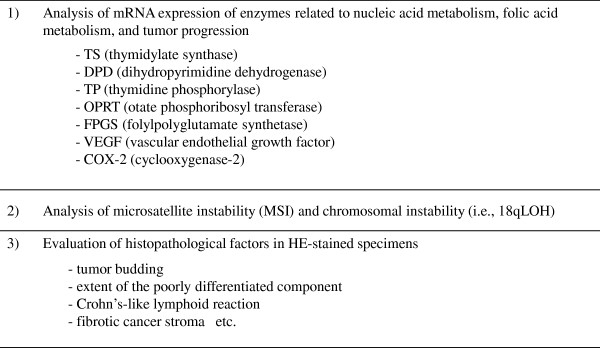
Items included in additional translational study.

The assessments shown in Figure 3 are made in paraffin-embedded thin sections of surgical specimens from primary tumors to evaluate the correlation with recurrences, survivals and AEs. The details of methods and analytical procedures will be reported separately.

### Ethical matters

This study is conducted in accordance with the “Declaration of Helsinki” and “Ethical Guidelines for Clinical Research,” and has been approved by the Institutional Review Boards of each participating institute. Written informed consent is obtained from all patients before enrollment.

## Discussion

This study is conducted to prospectively evaluate adjuvant chemotherapy for stage II colon cancer in terms of the efficacy, safety and feasibility in a large population.

According to the Japanese “Guidelines for the Treatment of Colorectal Cancer” [17] published by the Japanese Society for Cancer of the Colon and Rectum (JSCCR), adjuvant chemotherapy is recommended for stage III colorectal cancer. However, in line with the major Western guidelines [11-13], the JSCCR guidelines states that adjuvant chemotherapy for stage II colon cancer is considered for patients with a “high-risk factor of recurrence” after adequate informed consent, although the efficacy of adjuvant chemotherapy for stage II colon cancer is not clearly demonstrated and “high-risk stage II” is not clearly defined. No definite conclusion has been reached on this clinically important issue, probably for the following reasons: 1) large number of patients would be required to evaluate the efficacy of adjuvant chemotherapy for stage II colon cancer because of good surgical outcome; and 2) no high-quality RCT for stage II colon cancer alone has been conducted.

The SACURA trial is a RCT in patients with curatively resected stage II colon cancer, evaluating whether 1-year adjuvant treatment with UFT improves the DFS and OS compared with observation without adjuvant treatment (superiority study). Between October 2006 and July 2010, a total of 2,024 patients were enrolled from the 270 institutes. In Japan, complete mesocolic excision with central vascular ligation (D3 dissection) [17-19] is the standard surgery for colon cancer. The institutions which met the conditions that the member of the JSCCR, more than 80 colorectal cancer surgery each year and D3 dissection as routine surgery were selected for the study to insure the quality of the study.

In the present study, the observation group is used to investigate the clinicopathological high-risk factors for recurrence, and the UFT adjuvant therapy group is used to evaluate the effect of adjuvant therapy on the patients with those “high-risk factors”. These assessments will provide useful information to determine the indication of adjuvant therapy for patients with stage II colon cancer.

New reliable risk factors of recurrence other than routine items in histopathological examination are expected. The present study evaluates the following histopathological markers as promising prognostic factors for stage II colorectal cancer: tumor budding [20], extent of the poorly differentiated component [21], Crohn’s-like lymphoid reaction [22], and fibrotic cancer stroma [23]. This is the first study to evaluate those new possible prognostic histopathological markers prospectively using a large sample size.

In recent years, risk classification for recurrence/prognosis and prediction of efficacy to chemotherapy based on the biomolecular profiles are intensively studied. The meta-analysis reported that MSI-high stage II colorectal cancer was characterized by a lower recurrence rate and better prognosis, compared with MSI-low and microsatellite-stable stage II colorectal cancer [24]. On the other hand, the pooled analysis disclosed that adjuvant chemotherapy with 5-FU drugs for MSI-high colorectal cancer resulted in poorer OS than those of patients without the chemotherapy [25], indicating that MSI may be interesting as a predictor of efficacy to 5-FU based chemotherapy. Deletion or loss of heterozygosity (LOH) of the long arm of chromosome 18 (18q) is considered as an indicator of chromosomal instability [26,27], which can be related to carcinogenesis and tumor progression. In the PETACC-3 molecular study [28], both the univariate and multivariate analyses in 420 patients without adjuvant chemotherapy after surgery for stage II colon cancer revealed that 18qLOH was a significant factor for poor prognosis and that MSI-high was a significant factor for good prognosis. In the present study, MSI and 18qLOH are evaluated in more patients collected prospectively than those in the PETACC-3 study.

The efficacy and AEs of 5-FU drugs may be related to 5-FU-related enzymes in blood or tumor [29,30]. In Japan, several oral 5-FU drugs with differing mechanisms of action have been frequently used, but few prospective studies with a large sample size about this issue have been conducted. In the present study, the tumor mRNA expression levels of enzymes related to nucleic acid metabolism, folic acid metabolism, and tumor progression are measured to evaluate the correlation with the prognosis and AEs to identify predictors of efficacy and safety. In the future, it is expected that oral 5-FU drugs can be used in personalized ways based on differences in the appearance of these enzymes.

In conclusion, the SACURA trial is a large, multicenter phase III RCT intended to demonstrate the efficacy and safety of postoperative adjuvant therapy in patients with stage II colon cancer by showing the superiority of 1-year adjuvant treatment with UFT to observation without any adjuvant treatment. The results will identify 1) “high-risk stage II” colon cancer, 2) predictors of efficacy and AEs of adjuvant chemotherapy with 5-FU drugs and 3) subgroup benefited from adjuvant chemotherapy, and will contribute to establish an improved therapeutic strategy for stage II colon cancer.

## Abbreviations

AEs, Adverse events; MSI, Microsatellite instability; OS, Overall survival; RCTs, Randomized controlled trials; RFS, Recurrence-free survival; DFS, Disease-free survival; AST, Aspartate aminotransferase; ALT, Alanine aminotransferase; JSCCR, Japanese Society for Cancer of the Colon and Rectum; LOH, Loss of heterozygosity.

## Competing interest

SACURA trial (BRI_CC0501, BRI_CC0502) was conducted by “Foundation for Biomedical Research and Innovation, Translational Research Informatics Center” with funding from Taiho Pharmaceutical Co. Ltd., Japan.

MI has received consulting fees from Taiho Pharmaceutical Co. Ltd., Bristol-Myers Squibb and Merck Serono Co. Ltd; honoraria from Taiho, Chugai Pharmaceutical Co. Ltd., and Yakult Honsha Co. Ltd.

HM has received consulting fees from Otsuka Pharmaceutical Factory, Inc. and Sysmex Co.; honoraria from Taiho, Chugai, Yakult Honsha, Daiichi Sankyo Co. Ltd., Astellas Pharma Inc.

NT has received research funding from Taiho, Chugai, and Yakult Honsha; honoraria from Taiho, Chugai, Takeda Pharmaceutical Co. Ltd., Merck Serono.

YS has received honoraria from Taiho, Chugai and Yakult Honsha.

KT has received honoraria from Taiho and Takeda.

KK has received consulting fees from Taiho and Chugai; honoraria from Taiho, Chugai, Bristol-Myers, Merck Serono and Otsuka.

MW has received research funding from Taiho, Chugai, Yakult Honsha, Johnson and Johnson K. K. and Covidien Japan Co. Ltd.

YK has received honoraria from Taiho and Chugai.

H. Ueno has received honoraria from Taiho, Chugai, Yakult Honsha, Bristol-Myers and Daiichi Sankyo.

TI has received research funding from Taiho; honoraria from Chugai.

H. Uetake has received consulting fees, research funding, and honoraria from Taiho, Chugai, Takeda, Bristol-Myers and Merck Serono.

SM has no competing interest.

ST has received consulting fees from Taiho, Daiichi Sankyo and Solasia Pharma K. K.; research funding from Daiichi Sankyo, Yakult Honsha and Kureha Co. Ltd.; honoraria from Daiichi Sankyo.

KS has received consultant fees, research funding and honoraria from Taiho, Chugai, Takeda, Yakult Honsha, Daiichi Sankyo, Bristol-Myers, Merck Serono, and Pfizer Co. Ltd.

## Authors’ contributions

MI, as a task manager, participated in entire coordinating of the study, data collection, data analysis, data interpretation, and writing of the manuscript. HM, NT, YS, KT, KK, MW, YK, and KS, as a steering committee, participated in all phases of this study, including design and writing of the protocol, data collection, data analysis, data interpretation, and preparation of the manuscript. H. Ueno, TI, and H. Uetake, as a steering committee for additional translational study, carried out the molecular and pathological evaluation, and participated in all phases of this study, including design and writing of the protocol, data collection, data analysis, data interpretation and preparation of the manuscript. SM and ST, as a chief of statistical analysis, participated in statistical setting of study design and data analysis. All authors reviewed and approved the final manuscript.

## Authors’ information

No relevant information.

## Pre-publication history

The pre-publication history for this paper can be accessed here:

http://www.biomedcentral.com/1471-2407/12/281/prepub
